# Mutation prevalence tables for hereditary cancer derived from multigene panel testing

**DOI:** 10.1002/humu.24053

**Published:** 2020-07-09

**Authors:** Steven N. Hart, Eric C. Polley, Amal Yussuf, Siddhartha Yadav, David E. Goldgar, Chunling Hu, Holly LaDuca, Laura P. Smith, June Fujimoto, Shuwei Li, Fergus J. Couch, Jill S. Dolinsky

**Affiliations:** ^1^ Department of Health Sciences Research Mayo Clinic Rochester Minnesota; ^2^ Ambry Genetics Aliso Viejo California; ^3^ Department of Medical Oncology Mayo Clinic Rochester Minnesota; ^4^ Department of Dermatology University of Utah Salt Lake City Utah; ^5^ Department of Laboratory Medicine and Pathology Mayo Clinic Rochester Minnesota

**Keywords:** *BRCA1*, *BRCA2*, cancer mutation prevalence, carrier, mutation risk

## Abstract

Multigene panel testing for cancer predisposition mutations is becoming routine in clinical care. However, the gene content of panels offered by testing laboratories vary significantly, and data on mutation detection rates by gene and by the panel is limited, causing confusion among clinicians on which test to order. Using results from 147,994 multigene panel tests conducted at Ambry Genetics, we built an interactive prevalence tool to explore how differences in ethnicity, age of onset, and personal and family history of different cancers affect the prevalence of pathogenic mutations in 31 cancer predisposition genes, across various clinically available hereditary cancer gene panels. Over 13,000 mutation carriers were identified in this high‐risk population. Most were non‐Hispanic white (74%, *n *= 109,537), but also Black (*n * = 10,875), Ashkenazi Jewish (*n * = 10,464), Hispanic (*n*  = 10,028), and Asian (*n*  = 7,090). The most prevalent cancer types were breast (50%), ovarian (6.6%), and colorectal (4.7%), which is expected based on genetic testing guidelines and clinician referral for testing. The Hereditary Cancer Multi‐Gene Panel Prevalence Tool presented here can be used to provide insight into the prevalence of mutations on a per‐gene and per‐multigene panel basis, while conditioning on multiple custom phenotypic variables to include race and cancer type.

## Graphical abstract

Multigene panel testing for cancer predisposition mutations is becoming routine in clinical care; however, data on mutation detection rates by gene and by the panel is limited, causing confusion among clinicians on which test to order. Using results from 147,994 multigene panel tests conducted at Ambry Genetics, we built an interactive prevalence tool to explore how differences in ethnicity, age of onset, and personal and family history of different cancers affect the prevalence of pathogenic mutations in 31 cancer predisposition genes, across various clinically available hereditary cancer gene panels. The Hereditary Cancer Multi‐Gene Panel Prevalence Tool presented here can be used to provide insight into the prevalence of mutations on a per‐gene and per‐multigene panel basis, while conditioning on multiple custom phenotypic variables to include race and cancer type.

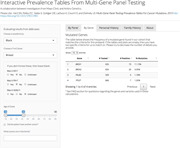

## 1 INTRODUCTION

Between 5% and 10% of all cancers are associated with an inherited mutation in a cancer predisposition gene. The high rate of mutations has led to a plethora of academic researchers and genetic testing laboratories focused on defining the risk and prevalence of mutations in multiple genes and how they are associated with various cancers. In an attempt to provide some guidance into who should be tested for predisposition mutations, the National Comprehensive Cancer Network (NCCN) set criteria to categorize individuals who are likely to contain a mutation in a predisposition gene—primarily based on an individual's personal and family history of cancers. However, recent data have demonstrated limitations in these selection criteria (Beitsch et al., 2019; LaDuca et al., 2019).

Historically, pretest probability models have been the gold standard to assess the likelihood that an individual is a mutation carrier in *BRCA1*/2. These include BOADICEA (Antoniou et al., 2008; Antoniou, Pharoah, Smith, & Easton, 2004), BRCAPRO (Biswas et al., 2013; Parmigiani, Berry, & Aguilar, 1998), the Myriad II (Frank et al., 1998; Frank et al., 2002), IBIS (Tyrer, Duffy, & Cuzick, 2004), Penn II (Couch et al., 1997; The Penn II Risk Model, BRCA 1 and BRCA 2 Mutation Predictor), and Manchester (Evans et al., 2004; Evans, Lalloo, Wallace, & Rahman, 2005) models for breast cancers and MMRpro (Chen et al., 2006) and PREMM (Kastrinos et al., 2011) for Lynch syndrome. All of these models were developed on relatively small patient populations (<10,000), and each their own unique limitations. More recently, Color Genomics released a website allowing quick perusal of genetic results from 50,000 individuals (Color Data Portal), with filtering criteria to better reflect the clinical characteristics of a given patient.

Here, we describe the development and demonstrate the functionality of an open‐access web‐based tool that allows the end‐user to query mutation prevalence across 49 genes and nine cancer indications with fine‐grained control of demographic and clinical history factors taken from 147,994 individuals.

## 2 DATA SPECIFICATION

**Table:** 

Data type	Interactive tables and figures
Data acquisition method	NGS
Data format	Analyzed
Experimental factors	147,994 Individuals referred to Ambry Genetics for hereditary cancer testing.
Experimental features	Data were formatted into a custom R DataFrame (v.3.3.3) object and loaded into an RShiny (v1.1.0) application. Filtering uses tidyverse (v.1.2.1), graphics with ggplot2 (v.2.3.1).
Data source location	NA
Data accessibility	The application is located at https://www.ambrygen.com/prevalence-tool.

## 3 IMPACT OF DATA

This web‐based tool represents data from 147,994 individuals referred to Ambry Genetics for hereditary cancer testing, which is an order of magnitude larger than most of the datasets used for previous models. It also contains the largest number of testing results for Asian, Black, and Hispanic populations.

While the Hereditary Cancer Multi‐Gene Panel Prevalence Tool was primarily designed to support clinical decision making, it could also serve as a useful resource for researchers interested in studying a specific cohort. This tool would aid investigators in the study design process by allowing them to analyze broad trends and assess feasibility based on the size of a given cohort. This tool allows the flexibility to search the parameters of interest in an appropriate cohort rather than relying only on data breakdowns that others have previously published or asking targeted questions to the owners of the cohort data. For example, the tool shows that in individuals under the age of 45, who had ER‐positive breast cancer as their first cancer, mutations in the *CHEK2* gene are found in 4.3% of non‐Hispanic whites compared to only 0.73% of Blacks. A researcher could assess whether the sample size by ethnicity is sufficient to address their research questions.

## 4 EXPERIMENTAL DESIGN, MATERIAL AND METHODS

**Figure 1:**
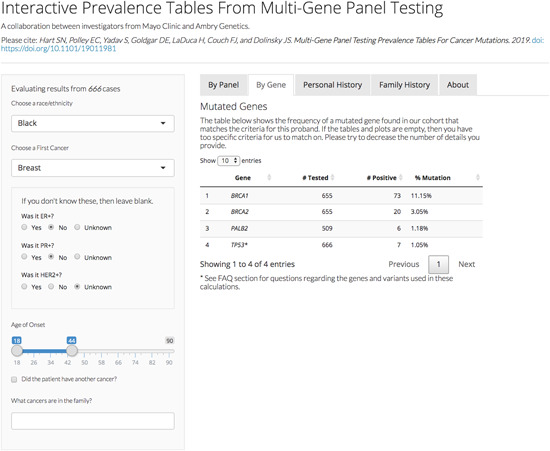
Screenshot of the interactive tool

Study subjects included patients who underwent multigene panel testing through Ambry Genetics (Aliso Viejo, CA) between March 2012 and December 2016. Individuals tested on the following panels were included BRCAplus®, BreastNext®, CancerNext‐Expanded®, CancerNext®, ColoNext®, GYNPlus®, OvaNext®, and PancNext®. Analysis of most genes on each panel consists of full gene sequencing of coding regions plus 5 base pairs into exon/intron boundaries (see Table 1) with some exceptions (LaDuca et al., 2019). Clinical histories were obtained from clinician‐completed test requisition forms (TRFs), along with clinical documentation such as pedigrees and clinic notes, when provided. Prior research has demonstrated a high level of accuracy of such clinical information provided on TRFs (LaDuca et al., 2018). This study was deemed exempt from review by Western Institutional Review Board. Personal and family histories for breast, colorectal, melanoma, ovarian, pancreatic, prostate, thyroid, reanl, gastric, leukemias, biliary, and uterine/endometrial were included if provided. Individuals were grouped into one of five racial and ethnic categories based on self‐report: and non‐Hispanic White, Black, Ashkenazi Jewish, Asian, or Hispanic (see Table 2). Only individuals between 18 and 90 years old are included. For breast cancer, data from estrogen receptor (ER), progesterone receptor (PR), and human epidermal growth factor receptor 2 (HER2) statuses were included where available. Pathogenic mutations include variants with a classification of “pathogenic” or “likely pathogenic” based on a five tier variant classification scheme (Pesaran et al., 2016). Data were formatted into a custom R DataFrame (v. 3.3.3) object and loaded into an RShiny (v1.1.0) application. Filtering uses tidyverse (v.1.2.1), graphics with ggplot2 (v. 2.3.1). The application is located at https://www.ambrygen.com/prevalence-tool (Figure 1).

**Table 1:** Panels and genes used in this study

Panel	Genes
BRCAplus	*BRCA1, BRCA2, CDH1, PALB2, PTEN, TP53*
BreastNext	*ATM, BARD1, BRCA1, BRCA2, BRIP1, CDH1, CHEK2, MRE11A, MUTYH, NBN, NF1, PALB2, PTEN, RAD50, RAD51C, RAD51D, TP53*
CancerNextExpanded	*APC, ATM, BAP1, BARD1, BRCA1, BRCA2, BRIP1, BMPR1A, CDH1, CDK4, CDKN2A, CHEK2, EPCAM, FH, FLCN, GREM1, MAX, MEN1, MET, MITF, MLH1, MRE11A, MSH2, MSH6, MUTYH, NBN, NF1, PALB2, PMS2, POLD1, POLE, PTEN, RAD50, RAD51C, RAD51D, RET, SDHA, SDHAF2, SDHB, SDHC, SDHD, SMAD4, SMARCA4, STK11, TMEM127, TP53, TSC1, TSC2, VHL*
CancerNext	*APC, ATM, BARD1, BRCA1, BRCA2, BRIP1, BMPR1A, CDH1, CDK4, CDKN2A, CHEK2, EPCAM, GREM1, MLH1, MRE11A, MSH2, MSH6, MUTYH, NBN, NF1, PALB2, PMS2, POLD1, POLE, PTEN, RAD50, RAD51C, RAD51D, SMAD4, SMARCA4, STK11, TP53*
ColoNext	*APC, BMPR1A, CDH1, CHEK2, EPCAM, GREM1, MLH1, MSH2, MSH6, MUTYH, PMS2, POLD1, POLE, PTEN, SMAD4, STK11, TP53*
GYNPlus	*BRCA1, BRCA2, BRIP1, EPCAM, MLH1, MSH2, MSH6, PALB2, PMS2, PTEN, RAD51C, RAD51D, TP53*
OvaNext	*ATM, BARD1, BRCA1, BRCA2, BRIP1, CDH1, CHEK2, EPCAM, MLH1, MRE11A, MSH2, MSH6, MUTYH, NBN, NF1, PALB2, PMS2, PTEN, RAD50, RAD51C, RAD51D, SMARCA4, STK11, TP53*
PancNext	*APC, ATM, BRCA1, BRCA2, CDKN2A, EPCAM, MLH1, MSH2, MSH6, PALB2, PMS2, STK11, TP53*

**Table 2:** Population demographics

	Non‐Hispanic white	Black	Ashkenazi Jewish	Hispanic	Asian	Total
	(*N* = 109,537)	(*N* = 10,875)	(*N* = 10,464)	(*N* = 10,028)	(*N* = 7,090)	(*N* = 147,994)
Breast						
Unaffected	51,341	4,042	5,538	4,816	2,868	68,605
Mean Age of Onset (SD)	50.3 (11.4)	47.2 (11.2)	52.6 (11.6)	46.2 (10.7)	45.6 (10.3)	49.7 (11.5)
Range	12.0–90.0	15.0–89.0	20.0–89.0	16.0–86.0	20.0–88.0	12.0–90.0
Ovarian						
Unaffected	100,551	10,396	9,964	9,410	6,494	136,815
Mean age of onset (SD)	57.3 (13.4)	54.5 (14.0)	58.8 (13.7)	51.8 (14.3)	52.3 (13.6)	56.7 (13.6)
Range	5.0–90.0	14.0–86.0	11.0–88.0	16.0–86.0	17.0–88.0	5.0–90.0
Colorectal						
Unaffected	103,169	10,268	10,112	9,467	6,729	139,745
Mean age of onset (SD)	50.0 (13.2)	47.6 (12.0)	52.6 (13.6)	45.5 (12.6)	45.2 (11.2)	49.4 (13.1)
Range	8.0–89.0	18.0–85.0	20.0–88.0	16.0–87.0	21.0–82.0	8.0–89.0
Uterine or endometrial						
Unaffected	105,734	10,651	10,167	9,725	6,892	143,169
Mean age of onset (SD)	54.3 (12.4)	52.9 (13.2)	57.4 (11.4)	47.5 (13.1)	48.9 (10.7)	53.8 (12.5)
Range	17.0–90.0	20.0–80.0	23.0–84.0	18.0–84.0	23.0–78.0	17.0–90.0
Pancreatic						
Unaffected	108,215	10,773	10,257	9,945	7,026	146,216
Mean age of onset (SD)	60.8 (11.6)	56.8 (11.9)	64.7 (11.0)	54.9 (12.9)	53.7 (14.7)	60.5 (12.0)
Range	20.0–89.0	26.0–80.0	31.0–88.0	22.0–82.0	9.0–83.0	9.0–89.0
Thyroid						
Unaffected	107,578	10,773	10,212	9,875	6,992	145,430
Mean age of onset (SD)	45.2 (13.8)	46.6 (12.2)	46.7 (13.8)	45.8 (13.3)	44.2 (11.4)	45.4 (13.7)
Range	8.0–89.0	21.0–78.0	6.0–75.0	16.0–81.0	14.0–74.0	6.0–89.0
Prostate						
Unaffected	108,841	10,826	10,362	10,004	7,077	147,110
Mean age of onset (SD)	59.9 (8.6)	58.9 (7.7)	62.6 (7.9)	61.0 (9.4)	63.2 (8.9)	60.2 (8.5)
Range	34.0–85.0	39.0–78.0	45.0–81.0	46.0–84.0	50.0–82.0	34.0–85.0
Kidney						
Unaffected	108,520	10,790	10,369	9,938	7,062	146,679
Mean age of onset (SD)	53.0 (14.8)	51.4 (14.3)	56.4 (12.2)	47.9 (12.3)	51.6 (11.0)	52.7 (14.4)
Range	1.0–87.0	6.0–77.0	27.0–79.0	2.0–74.0	31.0–74.0	1.0–87.0
Melanoma						
Unaffected	106,848	10,863	10,191	10,000	7,080	144,982
Mean age of onset (SD)	47.7 (14.4)	43.9 (16.2)	49.3 (14.6)	44.5 (14.9)	43.4 (15.3)	47.8 (14.5)
Range	1.5–90.0	19.0–69.0	3.0–90.0	21.0–73.0	18.0–69.0	1.5–90.0

## 5 DISCUSSION

As a demonstration of the utility of the tool, we posed the following question: “How different are mutation frequencies in the *MLH1* gene from colorectal cancer cases with a family history of pancreatic cancer versus the family history of prostate cancers?” To answer this question, the data were filtered for individuals with “First Cancer” as “Colorectal”, and then selecting either “Prostate” or “Pancreatic” in the box labeled “*What cancers are in the family?*”. After selecting the “By Gene” tab, the number of positive mutations and the number of tested per gene are returned for all genes, including *MLH1*. The numbers of individuals tested and positive are returned for all genes, including *MLH1*, which in this case was 26/845 (3.08%) in pancreatic cancer family histories versus 22/1477 (1.76%) with a family history of prostate cancer. Feeding these values into a Fisher's exact test confirm that pathogenic mutations were significantly higher in colorectal cases with a family history of pancreatic cancer (*p* = .0149).

### 5.1 Limitations of existing models

BOADICEA, BRCAPRO, Myriad II, IBIS, Penn II, and Manchester models for breast cancers are limited to the utility of predictions for *BRCA1* and *BRCA2*, as they are usually the only genes accounted for in these predictions due to the relatively low frequency of pathogenic mutations in other genes, however, BOADACEA now also provides a pretest probability for *ATM*, *PALB2*, and *CHEK2* mutations (Lee et al., 2019). These models were found to be reasonably accurate (Lindor et al., 2010), however, they were all derived from a small number of cases or families which may present bias. For example, the Penn II model was derived from 169 women of whom 16% were positive for *BRCA1* mutations. Manchester, BRCAPRO, and BOADICEA were developed from 1121, 2713, and 2785 probands or families, respectively, of which ~20% had pathogenic mutations in either *BRCA1* or *BRCA2*. The Myriad prevalence tables contain information from 10,000 consecutive cases through its clinical testing service; however, the data has not been updated since 2010, and thus may no longer be representative of the population referred for hereditary cancer testing today.

While they have been useful, a key limitation to all pretest probability models and existing prevalence tables/websites is the granularity at which they are published. The Myriad tables only contain two populations, Ashkenazi and non‐Ashkenazi Jewish. Family history information is limited to select combinations of breast and/or ovarian cancer personal and family history, even though there may be histories of other cancers. Some modeling tools can be overwhelmingly complicated or require downloading before running. If presented with insufficient numbers of exemplar data—or lack a strong statistical association for risk or outcome—then the model may not converge, failing to produce an accurate prediction.

Simpler, interactive tools are making mutation prevalence data significantly easier to access. In 2018, Color Genomics released a website allowing quick perusal of genetic results from 50,000 individuals (Color Data Portal). The user interface allows clinicians to estimate more refined mutation prevalence data using filtering criteria to better reflect the clinical characteristics of a given patient; however, the vast majority of tested individuals (n~40,000) do not have a personal history of cancer, which may limit the utility of this tool.

The Interactive Prevalence Tables From Multi‐Gene Panel Testing tool described here come with limitations as well, since ascertainment is based on a cohort of patients referred for hereditary cancer genetic testing due to clinical suspicion of hereditary cancer predisposition. Prevalence estimates may not be generalizable to the general population, but rather should be viewed in the context of the clinical and family history provided. The clinical and demographic data is limited to that provided to the researchers and testing laboratory, although such a limitation is a reality in any cohort represented in a pretest probability model. In addition, while the size of the cohort contributing to this tool is orders of magnitude higher than that in most other currently available pretest probability models or tools, greater numbers of patients are still needed, particularly for ethnic minority populations, genes in which mutations are rare, and queries for highly specific patient characteristics.

Despite these limitations, this tool is representative of patients referred for hereditary cancer panels and is therefore highly relevant to current genetic testing practices. Continued efforts to update this tool and others like it will provide continuous benefits to patients and providers by supplying relevant information in a timely manner. Thanks to large scale data sharing from commercial and academic entities, it is now possible to explore complex queries that more accurately reflect the clinical experience through a simple web‐based interface that draws upon data from large cohorts of patients recently referred for hereditary cancer multi‐gene panel testing.

## CONFLICT OF INTERESTS

Amal Yussuf, Holly LaDuca, Laura P. Smith, June Fujimoto, Shuwei Li, and Jill S. Dolinsky are all employees of Ambry Genetics.

## Data Availability

The application is located at https://www.ambrygen.com/prevalence-tool.
